# The Veterinary Medical Register

**Published:** 1881-07

**Authors:** 


The Veterinary Medical Register.—In response to a circular, published on another page, we have received a large number of names of veterinary practitioners. These have been arranged alphabetically, and are printed in the news department of the Journal. We are under obligations to a number of agricultural and medical journals for publishing our notice, and also to veterinarians for responding to it so promptly. It confirms us in our opinion of the usefulness of such a register.

The time allowed us for the compilation of the names already secured was too short to make the list complete. We shall, therefore, issue a supplementary list which will include all names sent to us after date of writing and before the next issue. The two lists will form a very complete register. We urge every veterinarian who has not done so to send his address, degree, if any, with the college which gave it, before the fifteenth of September next. To those who do thus send their names we will forward the list on receipt of three-cent stamps. Others can obtain them by forwarding nine cents for the first list and twelve cents for the two lists.

